# Gliding Arc Reactor under AC Pulsed Mode Operation: Spatial Performance Profile for NO*_x_* Synthesis

**DOI:** 10.1021/acssuschemeng.3c03832

**Published:** 2023-08-17

**Authors:** Sirui Li, Thijs van Raak, Rutger Kriek, Giulia De Felice, Fausto Gallucci

**Affiliations:** Inorganic Membranes and Membrane Reactors, Sustainable Process Engineering, Department of Chemical Engineering and Chemistry, Eindhoven University of Technology, De Rondom 70, Eindhoven 5612 AP, The Netherlands

**Keywords:** nonthermal plasma, nitrogen fixation, gliding arc reactor, pulsed mode operation, NO*_x_* synthesis

## Abstract

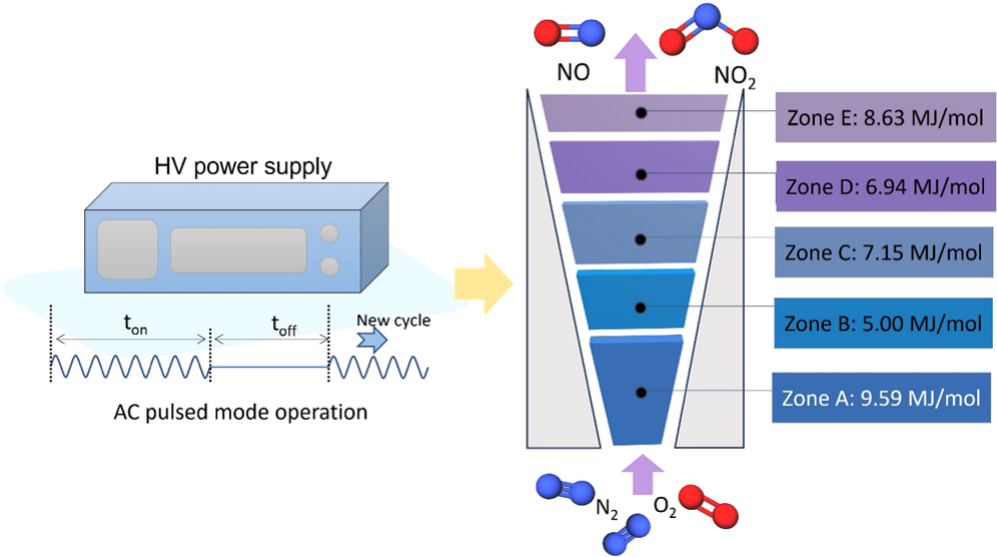

A two-dimensional gliding arc reactor for NO*_x_* synthesis was investigated in this study using AC pulsed mode operation. Tests with a duty cycle of 40 or 60% achieved the lowest energy consumption of 6.95 MJ/mol, which is an improvement of 15% from the case of continuous operation. Based on the results achieved, a new method for analyzing the spatial profile of the reactor was presented. The reactor was divided into five zones along the arc propagation, and results indicated that the first zone and last zone of the gliding arc reactor had higher energy consumption (9.59 and 8.63 MJ/mol, respectively), while lower consumption was observed in the middle parts of the reactor with a minimum of 5.00 MJ/mol. Spatial-resolved optical emission spectra, the deduced electron density, and temperature indicated the nonuniformity in plasma properties, which corresponds to the NO*_x_* production performance across the reactor. This research provides information and discussion that can be used for understanding and optimization of gliding arc reactors toward efficient nitrogen fixation.

## Abstract 2

The spatial profile analysis of a gliding arc reactor and the utilization of AC pulsed mode operation.

## 1 Introduction

The development of plasma technology, especially nonthermal plasma technology during the last few decades, has brought new opportunities for research in many different areas including CO_2_ conversion,^1−3^ material synthesis, and device fabrication^4−6^ as well as nitrogen fixation. Nonthermal plasma provides advantages such as mild reaction conditions and fast dynamic control and is suitable for small-scale production and utilization of renewable energy sources.^7^ It has been reported by many researchers that nonthermal plasma can be considered as a promising method for sustainable nitrogen fixation^8,9^ and a potential contributor to energy storage.^8^ Moreover, those advantages enable the on-demand, instant production of fixed nitrogen as a fertilizer, which meets the requirement for precision farming, bridging renewable energy with food production, and assisting the sustainable development of the world. Considering the application in fertilizer production, NO*_x_* synthesis by plasma process is more practical than ammonia production since air can be directly used as a feed gas and no hydrogen source is required. Besides, the theoretical energy consumption (EC) of plasma-based NO_x_ production can be even lower than the case of the Haber–Bosch process as suggested by Cherkasov et al.^10^ An efficient pathway for NO production can be provided in plasma through the vibrational excitation of N_2_, promoting the nonthermal Zeldovich mechanism as indicated in (1). This is followed by a chain reaction of the produced N reacting with O_2_ as shown in (2), resulting in a theoretical net energy consumption of 0.2 MJ/mol N for these two reactions.^11^ Previously, various studies of ammonia and NO*_x_* synthesis in plasma reactors have been reported including investigation on reactors,^12^ catalysts,^13,14^ reaction conditions,^12,13,16^ and kinetic analysis,^17,18^ as well as process development and evaluation^19^
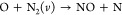
1
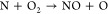
2Gliding arc is a “warm” type of nonthermal plasma with a gas temperature of up to a few thousand kelvin. Numerous studies have been published previously, covering topics ranging from the fundamentals of gliding arc^20^ to its various applications.^21−24^ It has advantages such as atmospheric operation, high flexibility, robust design, and high efficiency that can be achieved by using this type of reactor for chemical conversion.^25−27^ Wang et al. studied the mechanisms through chemical kinetics modeling and revealed that vibrational excitation makes a great contribution to N_2_ activation and results in the energy-efficient production of NO*_x_* in a gliding arc reactor.^28^ It should be noted that the gliding arc plasma discharge has a dynamic behavior as the discharge cyclically develops from the ignition, propagation, and to the cutoff stage. The plasma properties and other factors such as gas velocity and discharge gap could vary with this development. This brought nonuniformity of the reactor performance regarding the conversion, selectivity, and energy efficiency.

So far, most of the experimental studies evaluated the performance of gliding arc reactors as a whole, while the spatial profile has rarely been investigated. Besides, in most of the reported cases, only a continuous power supply mode was used for plasma generation and operating parameters such as flow rate and discharge frequency were often investigated to optimize the production of desired chemicals. Burst mode operation has been investigated by Ozkan et al. in a DBD reactor for CO_2_ conversion.^29^ However, such a kind of discontinuous power supply mode has not been explored in the case of NO*_x_* production with gliding arc reactors. In this study, the AC pulsed mode operation was investigated by using a gliding arc reactor. The spatial performance profile of the reactor along the direction of arc propagation has been analyzed and discussed, providing information for a better understanding of the plasma reactor design and further optimization.

## 2 Experimental Setup and Methodologies

**Figure 1:**
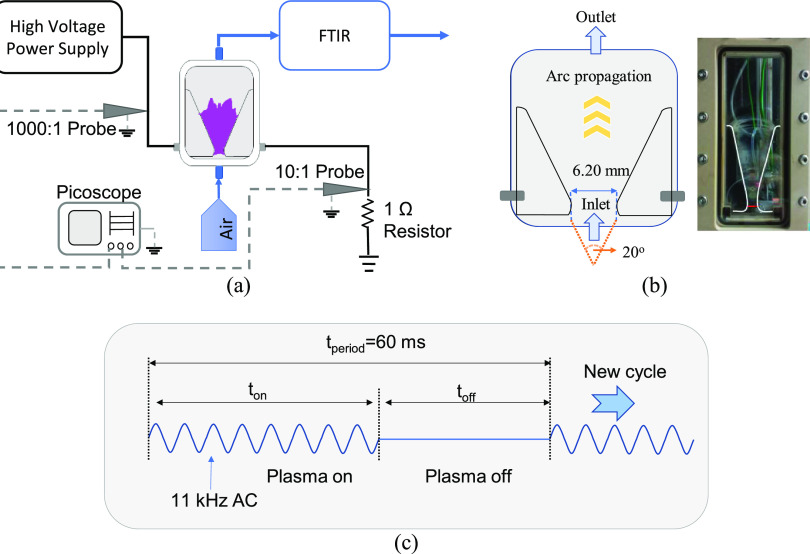
(a) Schematic diagram of the experimental setup, (b) gliding arc reactor, and (c) AC pulsed mode operation.

A scheme of the experimental setup used in this paper is shown in Figure 1a. Dry air was directly used as a feed gas in this series of experiments; the flow rate was set as 0.50 L/min by a mass flow controller (Bronkhorst). A Shimadzu IRTracer-100 Fourier transform infrared spectrophotometer (FTIR) equipped with a gas cell using a BaF_2_ window was connected to the outlet of the reactor to record the spectra of the product. Those spectra were analyzed with premade calibration curves by using Labsolution software to obtain the concentration of products. In our case, NO and NO_2_ are the main products; other products such as N_2_O and N_2_O_5_ were not detected. Therefore, NO*_x_* refers to the sum of NO and NO_2_ in this study. An AC high-voltage power supply (AFS G05F) was used for plasma generation, and it was connected to a waveform generator (SDG 1032X) for the control of the pulsed mode operation. A 1000:1 high-voltage probe (Tektronix P6015A) was used to measure the voltage across the reactor, and a 10:1 probe along with a current viewing resistor (1 Ω) was used to measure the current. Both voltage and current waveforms were recorded by a 5 Gs/s oscilloscope (Picoscope 6402D). The plasma reactor, as illustrated in Figure 1b, consists of a pair of bent tungsten wire electrodes with a thickness of 1.00 mm. The electrodes are symmetrically positioned, forming a discharge space with an angle of 20° between them and a narrowest gap of 6.20 mm. To enclose the electrodes and the discharge gap, two pieces of quartz glass are utilized, while a reactor case made of peek material provides structural support. The reactor case is securely sealed with screws and rubber gaskets to ensure a leak-free operation. Gas inlet and outlet ports are situated at the bottom and top of the reactor, respectively. Additionally, electrical connection points on either side of the reactor are established to connect the electrodes with the high-voltage power supply and the ground via the current viewing resistor.

To generate plasma, AC high voltage with a frequency of 11 kHz in pulsed mode was supplied to the gliding arc reactor. The setting of pulsed mode was done by adjusting the input signal from the waveform generator, which was used as a gating signal to switch the output of the power supply on and off accordingly as shown in Figure 1c. In this study, experiments were conducted by varying the duty cycle, which is defined as
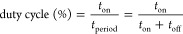
3where *t*_on_ and *t*_off_ are the time of plasma on and off, respectively. The total period *t*_period_ was set to 60 ms, which corresponds to one natural gliding arc cycle (duration from ignition to extinction) under continuous mode operation. When the pulsed mode was applied, the generation and propagation of the arc only occurred during the “on time” and was forced to stop during the “off time” period.

The discharge power was calculated based on the measurement of current and voltage across the reactor as shown in eq 4
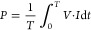
4The selectivity of NO and NO_2_ is calculated according to eqs 5a and 5b, where [NO] and [NO_2_] are the concentration of NO and NO_2_, respectively
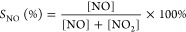
5a
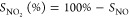
5bThe energy consumption (EC) for NO*_x_* production is defined as follows
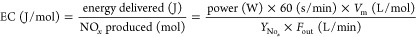
6where *P* is the discharge power calculated by eq 4. *V*_m_ is the molar volume of 24.0 L/mol since our measurement conditions in the FTIR are always 20 °C and 1 atm. Y_NO*_x_*_ is the volumetric fraction of NO*_x_*. *F*_out_ is the flow rate at the outlet of the reactor, which can be calculated from the known inlet flow rate with the consideration of the gas volume change caused by the reaction. It is important to acknowledge that the energy consumption discussed in this study specifically focuses on the gliding arc reactor. In practical applications, there is additional energy consumed in various components, including other parts of the circuit, gas system, absorption columns, and more. Therefore, when assessing the overall energy consumption of the process, it is crucial to consider these factors as well.

The emission spectra of the plasma were recorded using an optical emission spectrometer system (5 X AVASPEC-ULS4096CL-EVO-RS-RM-ET, AVANTES). The system comprises five channels for measurement, each with a resolution of ±0.1 nm. The integration times used for all five different channels and their respective wavelength ranges can be found in Table 1. The groove density and entry slit for every spectrometer were 1800 mm^–1^ and 10 μm, respectively. The optical probe was held by a robot arm (WLKATA Mirobot), allowing precise control of the measurement position based on the coordinates.

**Table 1:** Wavelength Range and Integration Time of the Five Channels

spectrometer	wavelength range (nm)	integration time (s)
1	200–367	4.4
2	367–512	3.5
3	512–634	2.0
4	634–733	8.0
5	733–811	0.9

The excitation temperature *T*_exc_ was calculated using the Boltzmann plot method, as shown in eq 7. Six lines from the N II system were selected, and their wavelengths (λ_ki_), corresponding transitions, statistical weight (*g*_k_), transition probability (*A*_ki_), and the energy of the upper level (*E*_k_) are listed in Table 2. The values were obtained from the NIST Atomic Spectra Database^30^
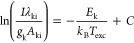
7

**Table 2:** Spectral Lines and Corresponding Data for the Calculation of *T*_exc_

λ_ki_ (nm)	transitions	*g*_k_	*A*_ki_ (s^–1^)	*E*_k_ (cm^–1^)
422.774	2s^2^2p4s ^1^P_1_^0^ → 2s^2^2p3p ^1^D_2_	3	1.08 × 10^8^	197,858.69
463.054	2s^2^2p3p ^3^P_2_ → 2s^2^2p3s ^3^P_2_^0^	5	7.48 × 10^7^	170,666.23
480.329	2s^2^2p3d ^3^D_3_^0^ → 2s^2^2p3p ^3^D_3_	7	3.17 × 10^7^	187,491.90
489.511	2s^2^2p3p ^1^P_1_ → 2s^2^2p^3 1^D_2_^0^	1	6.98 × 10^7^	188,937.24
500.515	2s^2^2p3d ^3^F_4_^0^ → 2s^2^2p3p ^3^D_3_	9	1.14 × 10^8^	186,652.49
504.510	2s^2^2p3p ^3^S_1_ → 2s^2^2p3s ^3^P_2_^0^	3	3.37 × 10^7^	168,892.21

The electron density was determined via the Stark broadening using eq 8a, which can be simplified by neglecting the ionic broadening, which is generally very small when considering an atomic line
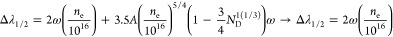
8a
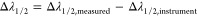
8bwhere Δλ_1/2_ is the full width at half-maximum (FWHM) of the spectral line in nm, and ω is the electron impact parameter. A is the ion broadening parameter, *n*_e_ is the electron density in cm^–3^, and *N*_D_ is the number of particles in the Debye sphere. Instrument broadening was measured via a Hg lamp and deducted from observed data as shown in eq 8b. The value of Δλ_1/2,instrument_ was 0.0942 nm in our case. The nitrogen spectral line at 746.8 nm was used in several reported studies and is also selected in this case.^31^ The corresponding electrical width parameter (ω) was 0.0299 nm.^32^

## 3 Results and Discussion

### 3.1 Effect of Duty Cycle

**Figure 2:**
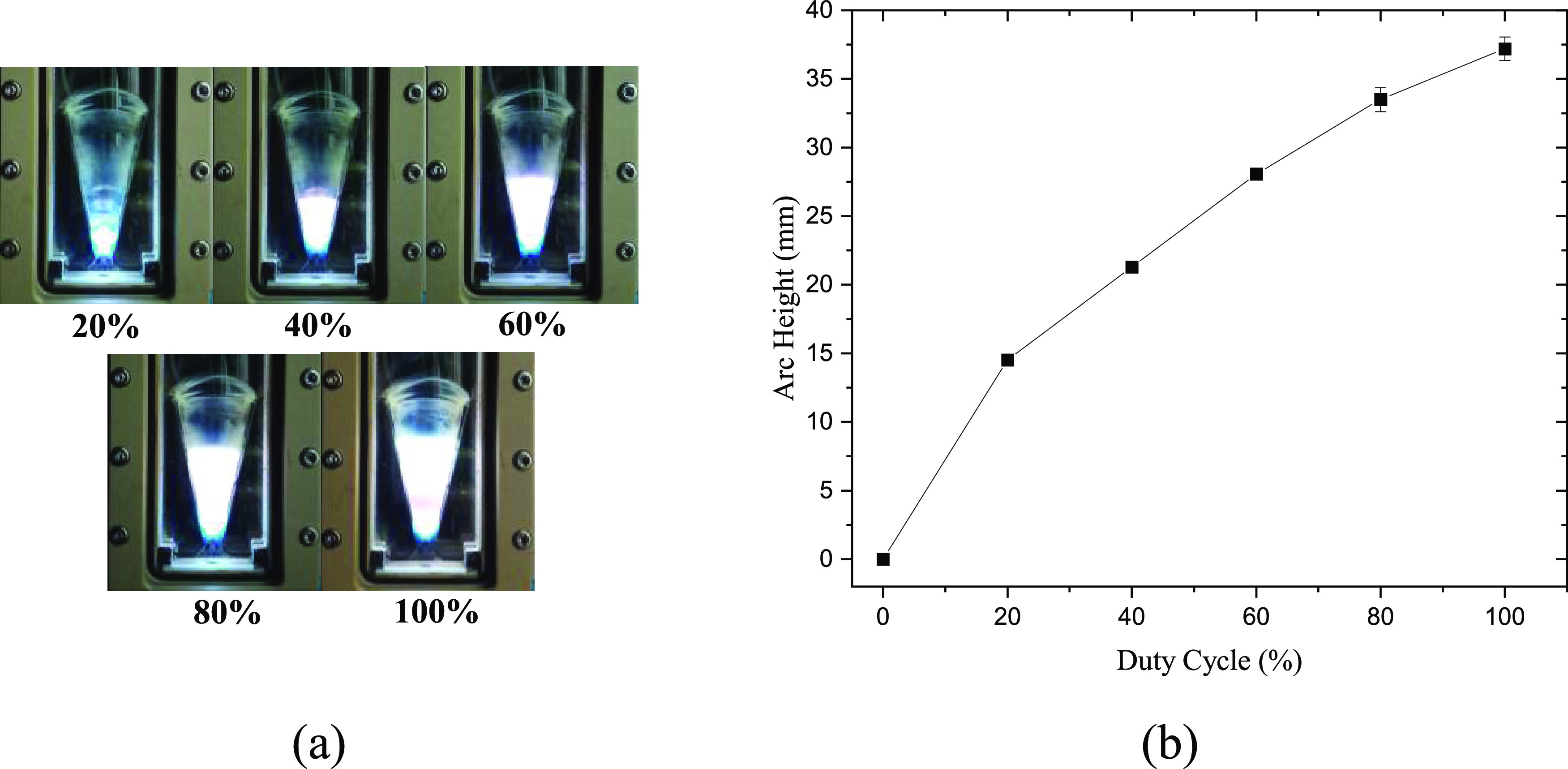
Variation of the gliding arc height with duty cycle: (a) illustration of the recorded pictures showing arc height under different duty cycles and (b) measured arc height as a function of the duty cycle.

The experiment was first conducted by varying the duty cycle from 0% (no plasma) to 100% (continuous mode) with an increment of 20%. The arc height was determined by using the pixel coordinates of the lowest and highest arc points on the recorded pictures, and the number increased with the duty cycle as shown in Figure 2. A larger duty cycle has longer “on time” for the power supply to sustain the plasma, allowing the arc to glide higher inside the reactor. In addition, the arc height is not strictly linear with the increase of the duty cycle. The increment in arc height became less significant with the increase in the duty cycle. This can be explained by a slower arc propagating at a higher section of the reactor. The gas velocity decreased at a higher position where the gap between electrodes is larger, leading to less force to drag the arc toward the top of the reactor. This is in line with several studies in which the highest gas velocity was reported at the narrowest gap.^33,34^ Besides the gas velocity, the influence of a thermal buoyant force could also play a role. Very often, the dominant role of a gas drag has been assumed in many reported studies due to the high flow rate used (several L/min to 10 L/min).^35,36^ In our case, a low flow rate was applied (0.5 L/min), but the discharge gap is also narrow (in the mm range). Hence, the influence of a thermal buoyant force on the arc height in our case is not clear and requires in-depth investigation. This may involve the study of temperature and density differences between the plasma channel and surrounding gas, which could be complex. However, several studies have been conducted to study the arc motion contributed by buoyant force under normal or hypergravity conditions.^37,38^ Further study with implementation of the approaches in those studies could be beneficial.

**Figure 3:**
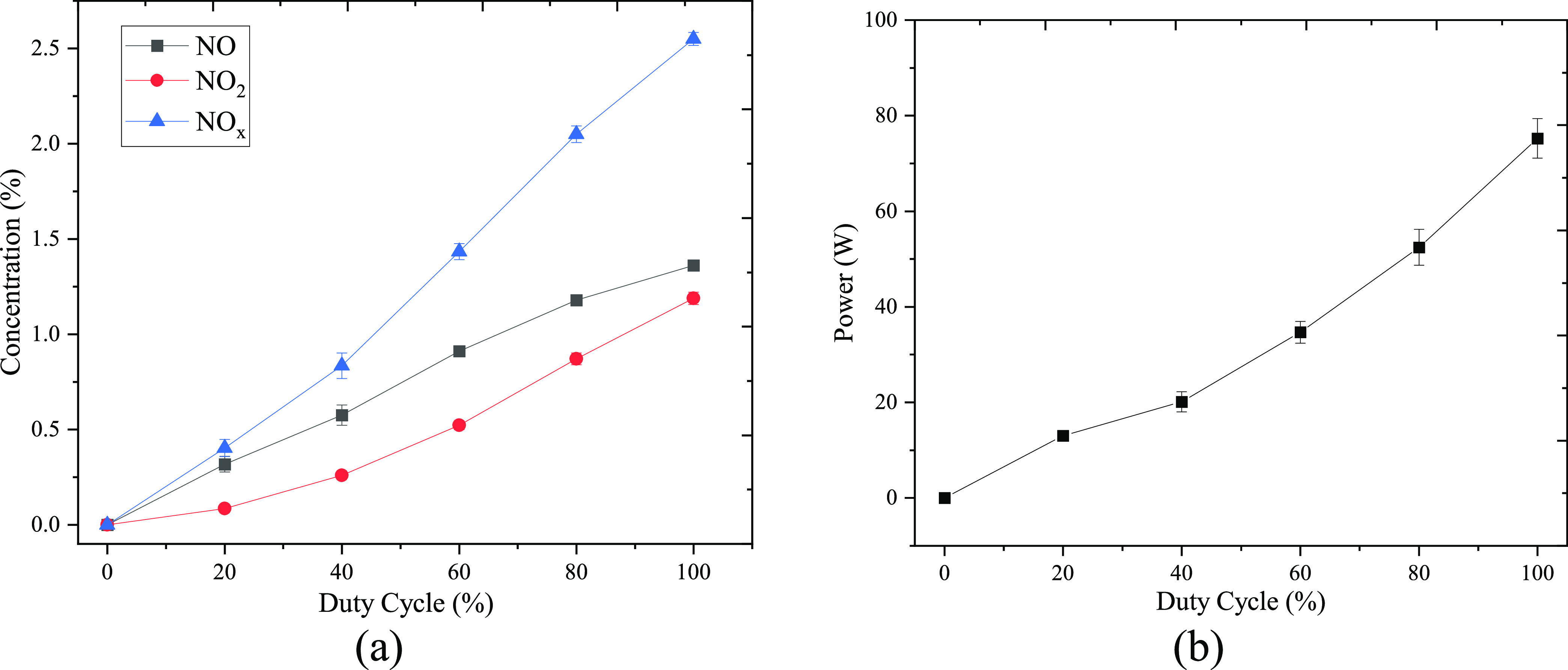
(a) Concentration of products and (b) discharge power as a function of duty cycle.

Higher concentration of both NO and NO_2_ in the outlet gas stream was observed in the cases with a higher duty cycle, as shown in Figure 3a. The maximum concentrations of NO, NO_2_, and NO*_x_* were achieved at a 100% duty cycle with 1.36, 1.18, and 2.55%, respectively. A higher duty cycle allows for a longer duration of plasma operation, resulting in higher power output, as shown in Figure 3b. This enables more energy to be delivered for NO*_x_* production, leading to increased concentrations of both NO and NO_2_. Furthermore, an increase in arc height was observed with a higher duty cycle, which resulted in a larger plasma volume for the gas to pass through in the reactor, resulting in a longer residence time for more NO*_x_* production. These two factors also contributed to the enhanced oxidation of NO to NO_2_, resulting in a higher selectivity of NO_2_.

**Figure 4:**
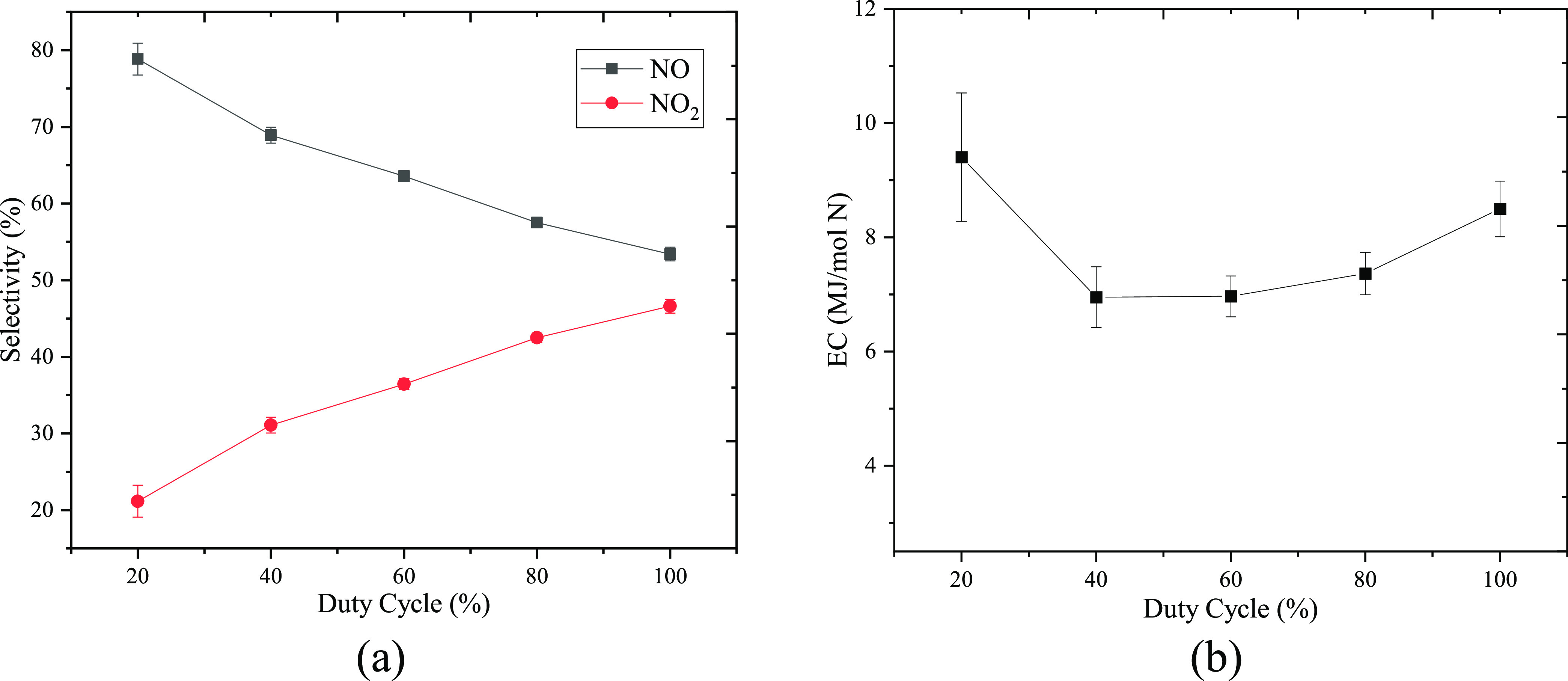
(a) Product selectivity and (b) energy consumption as a function of duty cycle.

As shown in Figure 4a, the highest selectivity of NO (78.69%) was achieved at the lowest duty cycle of 20%, and the selectivity decreased to 53.31% at a 100% duty cycle. More importantly, the energy consumption for NO*_x_* production varied with different duty cycles as shown in Figure 4b. Operation with a 20% duty cycle resulted in the highest EC with an average of 9.40 MJ/mol, while the EC in the case of continuous mode was 8.15 MJ/mol. It should be noted that the lowest EC (average ∼6.95 MJ/mol) was achieved with 40 and 60% duty cycles. These results demonstrated a potential way to reduce the energy consumption of gliding arc reactors by applying pulsed mode operation with a proper duty cycle. The observed behavior of EC is related to the nonuniformity of the reactor performance, which will be investigated in Section 3.2.

The Birkeland–Eyde thermal plasma process, established a century ago, demonstrated an energy consumption range of 2.4–3.1 MJ/mol for NO production with a concentration of approx. 2%.^10,39^ Recent research developments in plasma-based nitrogen fixation have shown significant promise, particularly with the implementation of nonthermal plasma technology, which exhibits an even lower theoretical minimum energy consumption. According to a technoeconomic analysis by Rouwenhorst et al., plasma NO*_x_* synthesis with an energy consumption of 2.4 MJ/mol N is almost competitive with the commercial process, while further reduction to 0.7 MJ/mol N would make it fully competitive.^11^ Reduction in energy consumption has been one of the primary targets for research on plasma nitrogen fixation. Table 3 presents an overview of recently published studies on NO*_x_* synthesis by plasma. Although a low EC (<1 MJ/mol) has been achieved in some cases, they are often associated with low product concentrations, leading to low production rates that hinder their application. It should be noted that the reactor design and operating parameters are of great importance. Examples can be found in research on rotating gliding arc reactors, which showed promising results: Van Alphen et al. developed an effusion nozzle, which improved the reactor performance,^40^ while Tsonev et al. achieved a high production rate of 69 g/h by operating the gliding reactor at an elevated pressure of 3 bars, with an energy consumption of 1.8 MJ/(mol N).^41^ In addition, utilizing a feed gas with an improved O_2_ content of up to 50% often results in a lower EC despite the potential increase in cost compared to using air alone. However, in our study, a normal gliding arc reactor was operated at atmospheric pressure with an air flow rate of 0.5 L/mol. We were able to achieve a 15% improvement in EC by only applying burst mode without any optimization of reactor design and operation parameters.

**Table 3:** Overview of Recently Published Studies in NO*_x_* Synthesis by Plasma

plasma/reactor	max conc. NO*_x_* (vol %)	EC (MJ/mol N)	flow rate (mL/min)	N_2_/O_2_ ratio	pres (atm)
DC glow discharge^15^	0.7	2.8	10,000	79:21	1
propeller arc^42^	0.45	4.2	300	2:1	1
Plasmatron^43^	1.5	3.6	10,000	70:30	1.23
MW plasma^44^	3.8	2.0	770	50:50	1
rotating gliding arc^45^	5.5	2.5	2,000	50:50	1
plasma jet/soft Jet^46^	0.02	0.42	1,500	79:21	1
rotating gliding arc^40^	5.9	2.1	2,000	50:50	1
rotating gliding arc^47^	0.28	0.67	170,000	79:21	1
rotating gliding arc^41^	4.9	1.8	12,000	50:50	3
this work	1.43	6.95	500	79:21	1

### 3.2 Spatial Performance Profile of the Gliding Arc Reactor

**Figure 5:**
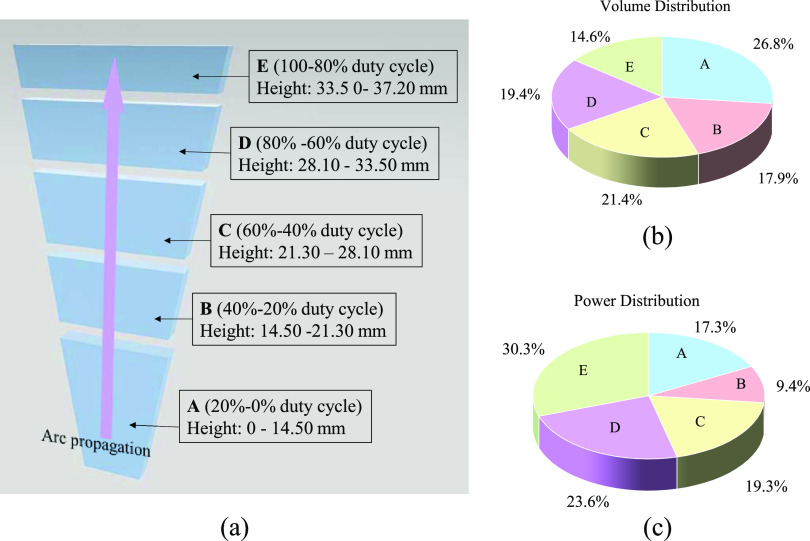
Spatial profile analysis of the gliding arc reactor: (a) zone division of the reactor from A to E, representing five distinct regions along the reactor length; (b) volume distribution among the divided zones; and (c) power distribution among the divided zones.

Under continuous mode operation, a natural single cycle of gliding arc consists of the ignition, propagation, and extinction within a period of 60 ms. Since the period of pulsed operation was set to a duration of 60 ms, a higher duty cycle can be interpreted as a lower duty cycle with an extended “on time”. Consequently, plasma generated with a higher duty cycle will not only contain the plasma that could be produced with a lower duty cycle but also contain additional plasma created during the extended “on time”. Based on this, the reactor can be divided into five zones as shown in Figure 5. Zone A encompasses the section of the reactor where the arc propagation occurs from a height of 0–14.50 mm, achieved through operation at a 20% duty cycle. Zone B encompasses the section with plasma heights from 14.50 to 21.30 mm, which correspond to the plasma heights for 20 and 40% duty cycle operations, respectively. This region can be seen as a part of the reactor that experiences further plasma propagation from the end of the 20% duty cycle to the end of the 40% duty cycle. The net NO*_x_* production rate in zone B can be determined by subtracting the 20% duty cycle value from the 40% duty cycle value. Subsequently, zones C–E were defined, and their net NO*_x_* production rate was obtained. It is important to highlight that by adjusting the duty cycle, the arc height can be controlled, allowing for the desired arc height to be achieved. This flexibility enables the vertical division of the reactor according to specific requirements. However, it is crucial to select a duty cycle increment that is sufficiently large to ensure the accurate measurement of the arc height, thereby reducing the margin of error and facilitating proper zone division. In this study, we utilized a step size of 20% duty cycle increments, which proved to be effective in achieving the desired level of precision in dividing the zones within the reactor.

By evaluating the performance of each zone, the spatial profile of the gliding arc was achieved. The volume of each zone can be calculated by taking into account the fixed depth of the reactor (1 mm) and the corresponding gap width between electrodes at the beginning and end of the zone. Considering the reactor working in continuous mode as a base case with a discharge power of 75.25 W with a total volume of 474.65 mm^3^, the power and volume distribution among the different zones can be calculated by dividing their respective values by the base case. The average results are shown in Figure 5, and the error margin is less than 5%. Zone E exhibits the highest power but the smallest volume, whereas zone A has the largest volume but the second smallest power.

**Figure 6:**
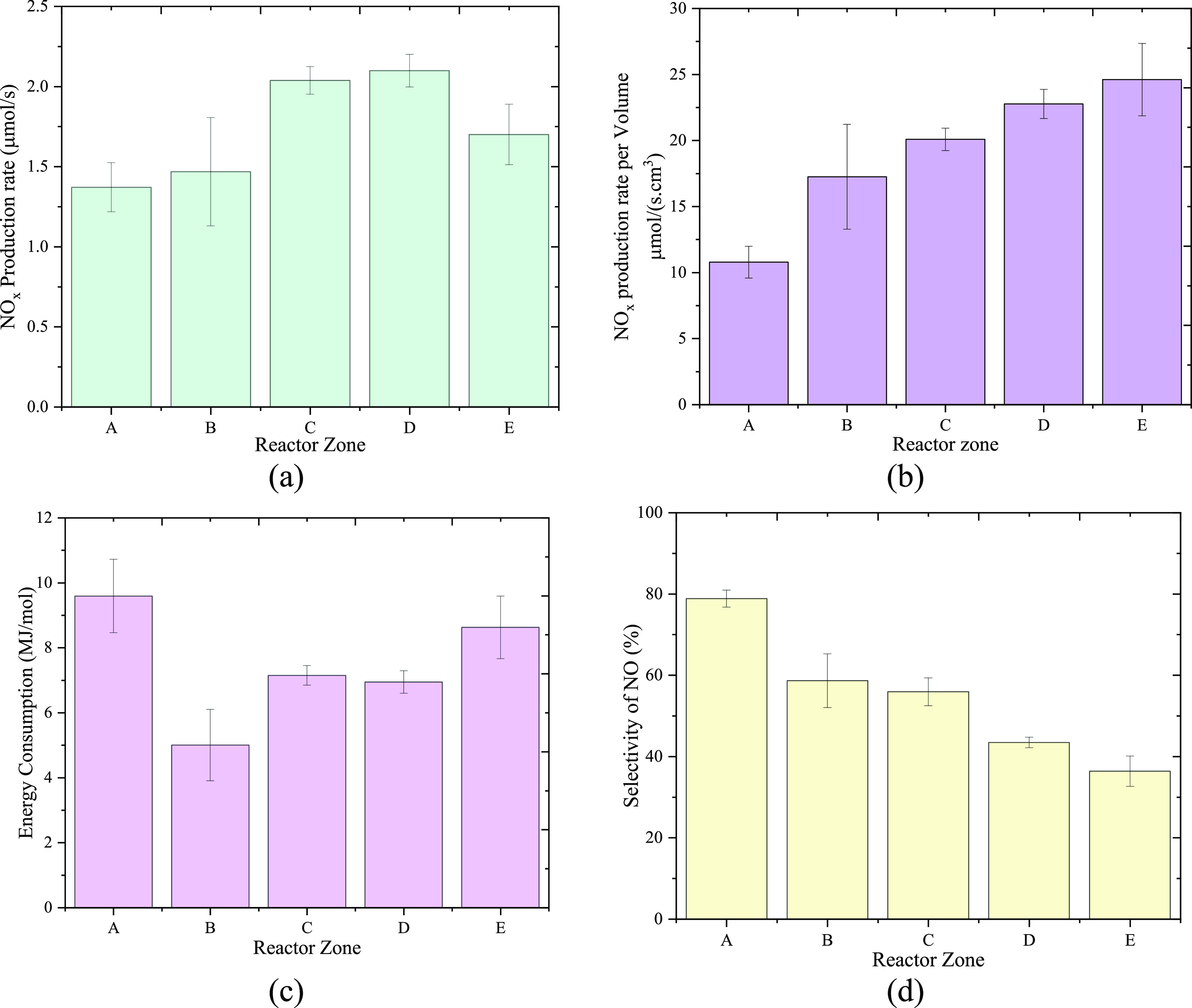
In each zone of the reactor: (a) NO*_x_* production rate, (b) NO*_x_* production rate per volume, (c) energy consumption, and (d) selectivity of NO.

The net NO*_x_* production rate of each zone is shown in Figure 6a. The highest production rate was observed in zone D, closely followed by the rate in zone C, indicating that these two zones are the most efficient sections of the reactor. It should be noted that the difference in the volume of zones influenced the production rate. Figure 6b shows the production per volume of each zone, and a clear upward trend from zone A to zone E can be seen, indicating a growing production rate of NO*_x_* per volume along the arc propagation direction. It should be noted that zone E has the highest production rate per volume, but the overall production rate is lower than C and D. One of the main reasons is the smallest volume of zone E, which corresponds to a low residence time that limited the overall production despite the highest power delivered.

**Figure 7:**
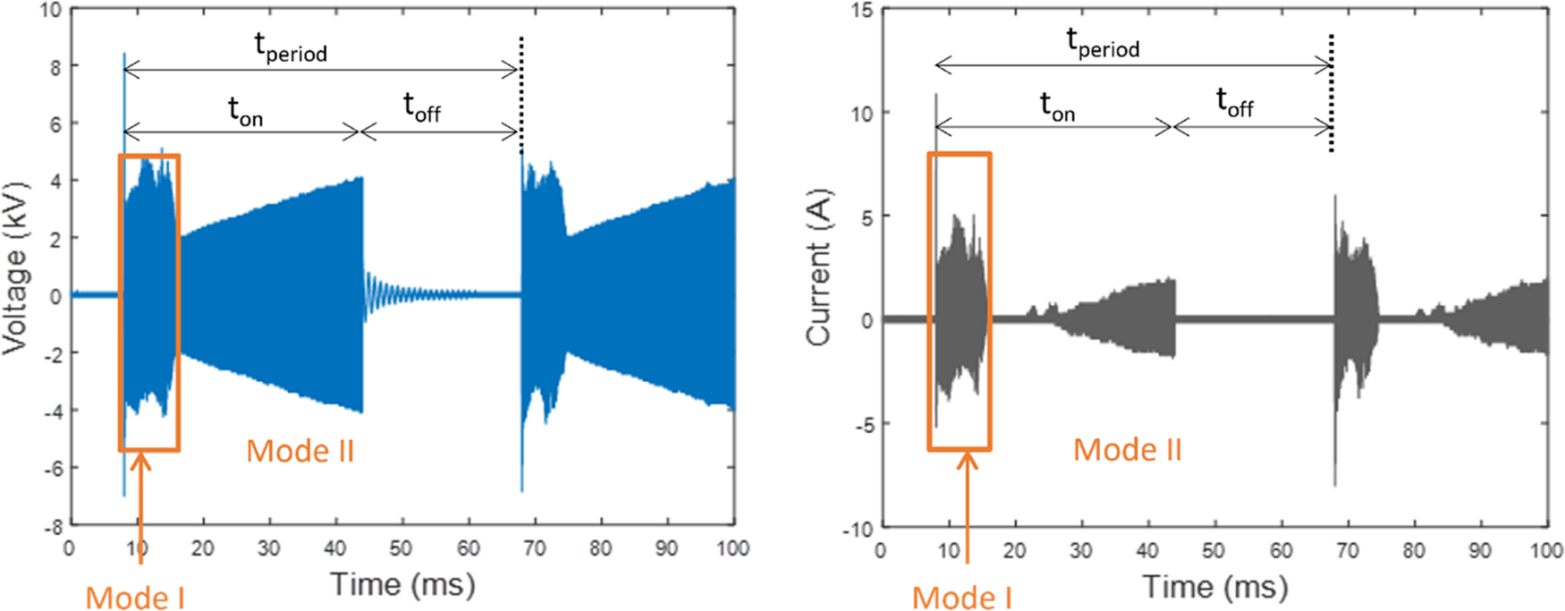
Example of current and voltage waveforms under a 60% duty cycle.

The energy consumption of NO*_x_* production in each zone is shown in Figure 6c. The beginning and near the end of the gliding arc plasma (represented by zones A and E, respectively) had higher energy consumption, while lower consumption was observed in the middle zones of the reactor. In the case of zone A, the highest EC was observed. It is worth mentioning that the discharge power in zone A is not in line with the general increasing trend from zone B to zone E across the reactor. This is mainly caused by the discharge characteristics, which can be reflected in the voltage and current waveforms as in Figure 7. At the beginning of a gliding arc cycle, high peaks in current and voltage were always observed, and this part of discharge is in a high-voltage breakdown mode (mode I), which has also been reported by Cheng et al.^48^ In mode I, high power was delivered by the power supply to trigger the initial ignition of the arc within the narrow gaps at the bottom of the reactor. The discharges in this mode have a similar behavior as a static arc, which has different plasma properties than general gliding arcs. Subsequently, under the influence of gas flow, the arc exhibited continuous rotation and elongation, ultimately transforming into a gliding arc in mode II. In this mode, the arc initiation involved a relatively low current in the range of tens of mA, as indicated by the waveform measurements. However, as the process progressed, the current underwent a transition, leading to the emergence of high current peaks reaching a few Amperes at a later stage. The high energy consumption in zone A can be attributed to the presence of “mode I”. While both zone A and zone E experience high energy consumption, zone E can be “deactivated” by using a pulsed mode operation with a duty cycle lower than 80%, thus reducing the overall energy consumption of the gliding arc reactor. On the other hand, zone A cannot be avoided since it serves as the foundation for the development of plasma in the other zones.

In addition, the selectivity of NO decreased from zone A to E as shown in Figure 6d. This indicates that more oxidation of NO to NO_2_ by oxygen species occurs along the arc propagation direction. It should be noted that NO could be oxidized by O_2_ since the reaction is spontaneous at ambient conditions. Therefore, part of the produced NO is converted to NO_2_ after it exits the plasma zone and travels through the pipelines before entering the gas cell of the FTIR. This may result in a generally lower measured NO selectivity in our case. To minimize this potential influence, a short pipeline (0.13 L) with a corresponding residence time of 15 s was used. However, based on our observations, this effect does not significantly impact the observed tendencies, which are primarily attributed to the plasma reactions. Plasma-generated reactive oxygen species play a major role in the oxidation of NO, which caused the difference in selectivity presented. According to Wang et al.,^28^ the vibrational excitation stimulated the Zeldovich mechanism as shown in (1), and is the dominating process for NO production, while the oxidation of NO by O generated by plasma through (9) is the most important pathway for NO_2_ production in gliding arc
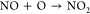
9

### 3.3 Spatially Resolved Optical Emission Spectra

**Figure 8:**
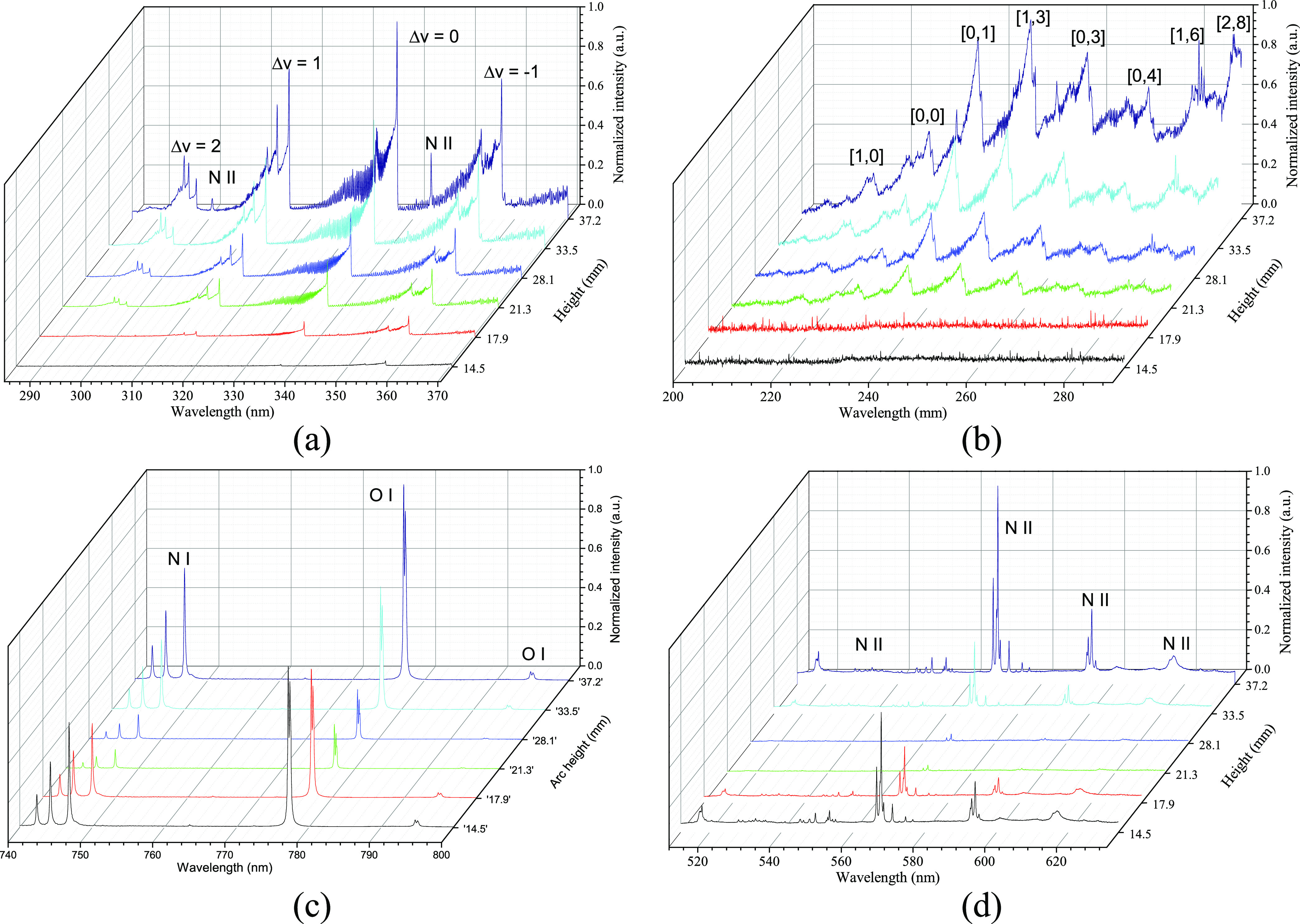
Optical emission spectra recorded at different heights with (a) nitrogen second positive system; (b) NO γ band; (c) atomic peaks of nitrogen and oxygen; and (d) ionized nitrogen N II.

To further investigate the properties of plasma in different regions of the reactor, spatially resolved optical emission spectra were recorded. The gliding arc reactor was operated under the continuous mode, and the measurement location was precisely controlled by setting the coordinates of the robot arm, which holds the optical probe. The measurement locations are positioned at heights of 14.5, 21.3, 28.1, 33.5, and 37.2 mm, which correspond to the arc heights observed in a pulsed mode with different duty cycles. Additionally, an extra measurement was taken at 17.9 mm in addition to the measurement at 14.5 mm, which exhibits low intensity due to the small discharge gap. A clear increase of intensity with height can be seen in peaks of bands including Δ = 2, 1, 0, and −1 in the nitrogen second positive system (SPS), as shown in Figure 8a. One important reason is the increased plasma volume, which is a result of the increased gap size with height. As for the NO γ band, a similar trend was observed in the spectra as shown in Figure 8b. The increasing intensity also relates to the increasing NO concentration across the reactor because of more produced NO by plasma. However, peaks were only observed from the third coordinate although NO is also produced at a lower region of the reactor. The peaks of ionized atomic nitrogen (N II) at 300.68 nm (4s^1^P^0^ → 3p^1^P) and 343.61 nm (3p^1^S^0^ → 3s^1^P^0^) were also observed with very low intensity at a height of 33.5 mm and became more obvious at 37.2 mm. A very different behavior was observed in the higher-wavelength range with the presence of atomic peaks of nitrogen and oxygen (N I and O I) as well as ionized nitrogen (N II) as shown in Figure 8c,d. This behavior is similar to the case of energy cost for NO*_x_* production, which also has a lower value in the middle zones of the reactor. The low intensity of atomic N and O peaks suggests a low concentration of these species, likely due to consumption in reactions that form NO*_x_*, and less dissociation of the formed NO*_x_*. This may help to explain why the middle section of the reactor has a higher production rate with lower energy consumption. A plausible mechanism can be proposed based on the observed results: at the lower section of the reactor where the gas enters, the dissociation of N_2_ and O_2_ occurs, leading to the production of atomic N and O species; hence, a high intensity of their peaks is observed in the emission spectra. The production of O is particularly important for the synthesis of NO*_x_* as indicated in eqs 7 and 8. In the middle section, the low intensity of atomic N and O peaks suggests a low concentration of these species, likely due to consumption in reactions that form NO*_x_*, as well as less dissociation of the formed NO*_x_*. This may help to explain why the middle section of the reactor has a higher production rate with lower energy consumption. Later, when the gas with an accumulated concentration of NO*_x_* enters the higher section of the reactor, plasma-induced reactions that dissociate NO*_x_* take place, leading to the production of N and O; hence, the peak intensity of atomic N and O species increases. This also leads to the relatively low production of NO*_x_* and high energy consumption as observed. However, the underlying mechanisms are rather complex, and details on the production and consumption rate of atomic species need to be further investigated.

**Figure 9:**
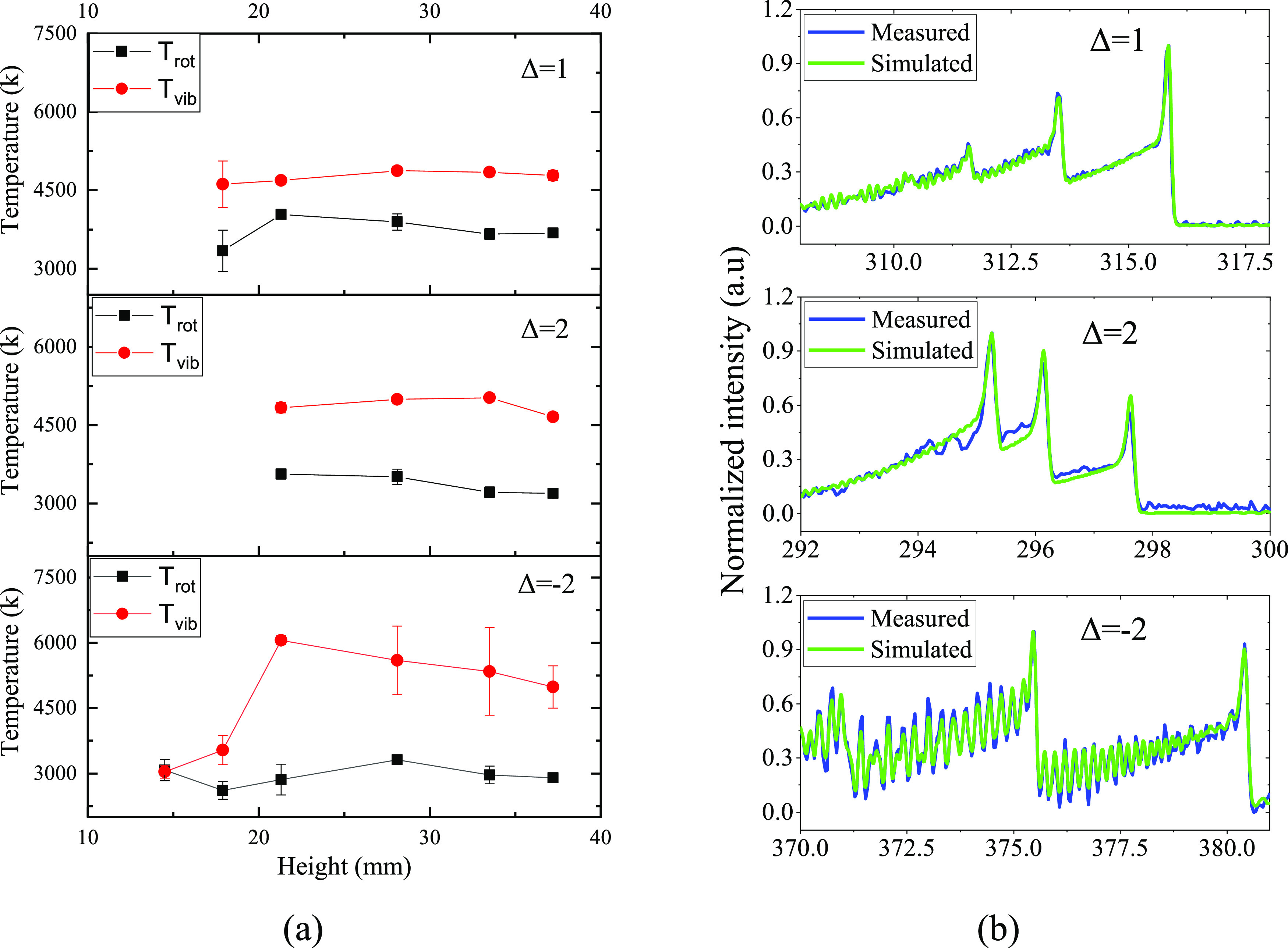
Vibrational and rotational temperatures obtained by fitting Δ = 1, 2, and −2 bands to simulated spectra from Specair: (a) *T*_rot_ and *T*_vib_ as a function of height and (b) examples of measured spectra fitted to simulated spectra.

To obtain information on the vibrational and rotational temperatures, the recorded spectra were fitted with simulated spectra using Specair software. The vibrational temperature obtained from OES in this study is not associated with the ground-state molecules but rather with the electronically excited molecules. Reasonable fittings were achieved with N_2_ SPS Δ = 2 and 1, and the corresponding temperatures are shown in Figure 9. The presented results are temperatures averaged in time and across triplet measurements. It should be mentioned that the temperature achieved by fitting different bands deviates from each other. Such a deviation was also observed in other studies.^49^ However, results from both bands showed a higher vibrational temperature than the rotational temperature above 20 mm, indicating a strong nonthermal equilibrium of plasma in those regions. Unfortunately, the temperature profile under a 20 mm height cannot be deduced due to the low intensity of peaks on spectra. Spectra with N_2_ SPS Δ = −2 were also fitted due to their strong peaks in this good resolved rovibrational band.^49,50^ In this case, the temperature profile at all tested heights was obtained and the results are shown in Figure 9a. At the lowest height, the vibrational temperature and rotational temperature are very close; this can be linked to the arc under mode A, which has different plasma properties.

**Figure 10:**
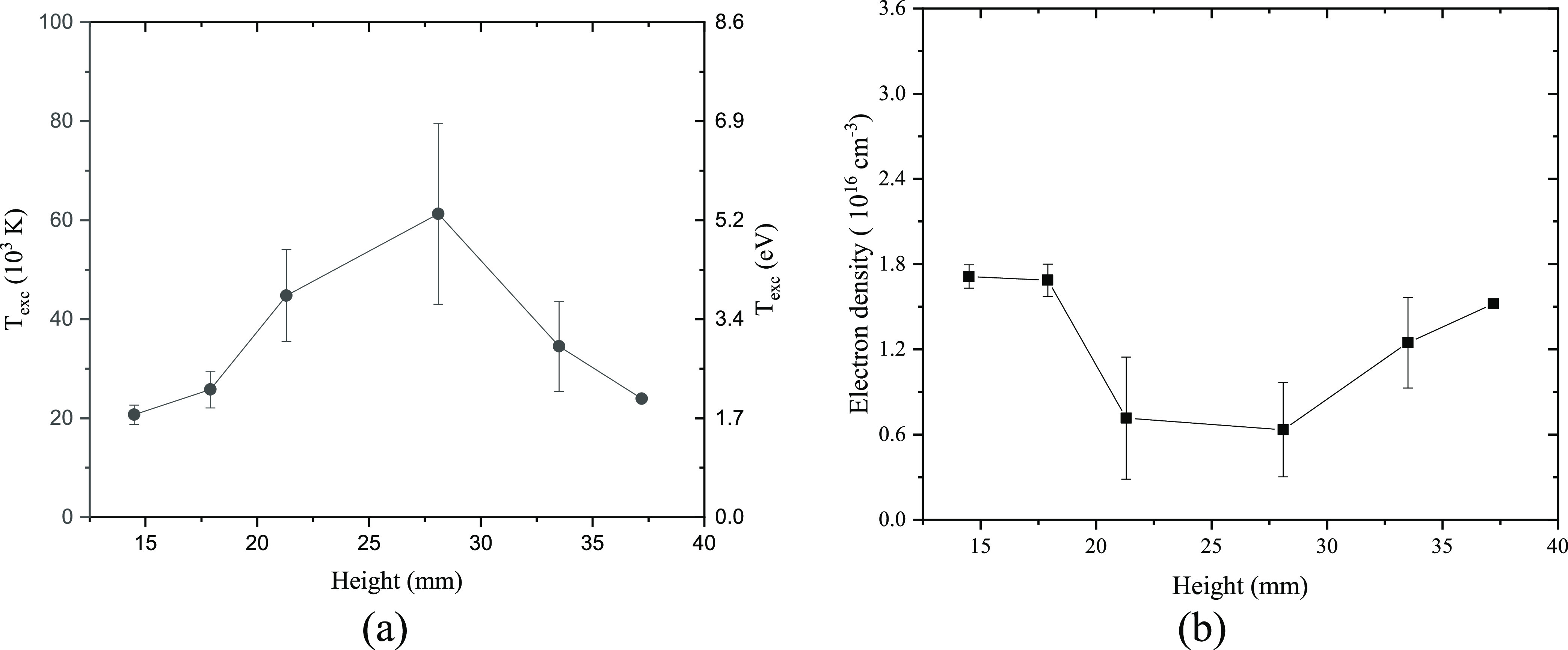
(a) Excitation temperature and (b) electron density as a function of height. Spectra for evaluation were measured at different heights across the reactor under continuous operation.

The excitation temperature at different heights was calculated using the Boltzmann plot method, and the results are presented in Figure 10a. The calculated excitation temperature was found to be higher than the vibrational and rotational temperatures, irrespective of the height variation. This suggests a nonthermal equilibrium across the reactor. Moreover, a higher *T*_exc_ was observed in the middle section of the reactor, with an average value exceeding 6 eV, while the excitation temperature at the top and bottom of the reactor was below 3 eV. As for the calculation of the electron density, an opposite parabolic behavior was observed with low values in the middle part of the reactor as shown in Figure 10b. This corresponds to the current waveform depicted in Figure 7. In mode I, occurring at the beginning of the gliding arc in the lower section of the reactor, high currents were observed. Subsequently, in mode II, the current initially remained low but gradually increased at a later stage.

In addition, this behavior of electron density also refers to the intensities of nitrogen and oxygen peaks observed in Figure 8c,d. High electron density in the lower and higher regions of the reactor is favorable for the dissociation of N_2_ and O_2_. However, the dissociation of formed NO*_x_* negatively impacts the reactor performance, which may be the reason for the similar parabolic trend observed in energy consumption in Figure 6c. While the effect of the variation in electron density and excitation temperature across the reactor requires further exploration, accounting for detailed reaction mechanisms, it is evident that the nonuniformity of plasma properties has a strong correlation with the spatial performance profile of the reactor. This finding highlighted the critical role of achieving spatial uniformity in plasma properties to optimize reactor performance and enhance the efficiency of plasma-based processes. Further studies are needed to better understand the underlying mechanisms and establish a more comprehensive understanding of the complex interplay between plasma properties and reactor performance.

## 4 Conclusions

In this work, AC pulsed mode operation was investigated using a gliding arc reactor for NO*_x_* production. Results indicated that compared with continuous operation, improvement in energy efficiency can be achieved by applying a duty cycle of 40 or 60%. The main reason is the nonuniform properties of the reactor along the direction of arc propagation. Instead of studying the reactor as a whole, an analysis of the spatial profile was conducted by dividing the reactors into different zones. The middle part of the reactor showed lower energy consumption than the zones that contain arc ignition and extinction. The optical emission spectra recorded have also illustrated different properties of plasma at different locations inside the reactor, which corresponds to the production of NO*_x_* observed. The information provided by this study along with the methodology can be used for further research on the diagnostics and design of gliding arc plasma reactors.
